# Protein-lipid interactions: correlation of a predictive algorithm for lipid-binding sites with three-dimensional structural data

**DOI:** 10.1186/1742-4682-3-17

**Published:** 2006-03-28

**Authors:** David L Scott, Gerold Diez, Wolfgang H Goldmann

**Affiliations:** 1 Renal Unit, Leukocyte Biology & Inflammation Program, Structural Biology Program and the Massachusetts General Hospital/Harvard Medical School, 149 13^th ^Street, Charlestown, MA 02129, USA; 2 Friedrich-Alexander-University of Erlangen-Nuremberg, Center for Medical Physics and Technology, Biophysics Group, Henkestrasse 91, 91052 Erlangen, Germany

## Abstract

**Background:**

Over the past decade our laboratory has focused on understanding how soluble cytoskeleton-associated proteins interact with membranes and other lipid aggregates. Many protein domains mediating specific cell membrane interactions appear by fluorescence microscopy and other precision techniques to be partially inserted into the lipid bilayer. It is unclear whether these protein-lipid-interactions are dependent on shared protein motifs or unique regional physiochemistry, or are due to more global characteristics of the protein.

**Results:**

We have developed a novel computational program that predicts a protein's lipid-binding site(s) from primary sequence data. Hydrophobic labeling, Fourier transform infrared spectroscopy (FTIR), film balance, T-jump, CD spectroscopy and calorimetry experiments confirm that the interfaces predicted for several key cytoskeletal proteins (alpha-actinin, Arp2, CapZ, talin and vinculin) partially insert into lipid aggregates. The validity of these predictions is supported by an analysis of the available three-dimensional structural data. The lipid interfaces predicted by our algorithm generally contain energetically favorable secondary structures (e.g., an amphipathic alpha-helix flanked by a flexible hinge or loop region), are solvent-exposed in the intact protein, and possess favorable local or global electrostatic properties.

**Conclusion:**

At present, there are few reliable methods to determine the region of a protein that mediates biologically important interactions with lipids or lipid aggregates. Our matrix-based algorithm predicts lipid interaction sites that are consistent with the available biochemical and structural data. To determine whether these sites are indeed correctly identified, and whether use of the algorithm can be safely extended to other classes of proteins, will require further mapping of these sites, including genetic manipulation and/or targeted crystallography.

## Background

Signal transduction, vesicle trafficking, retroviral assembly, and other central biological processes involve the directed binding of proteins to membranes. Soluble proteins may associate with membranes through well-defined structural domains (e.g., pleckstrin-homology, PX (phox), C2, amphipathic helices and/or unstructured motifs that interact through non-specific electrostatic and non-polar interactions [1-3]. Post-translational modifications, such as myristylation or palmitoylation, may also play critical roles in regulating membrane association.

Many cytoskeleton-associated proteins interact, at least transiently, with membranes [4-6]. The application of biophysical techniques including Fourier-transformed infrared spectroscopy (FTIR), neutron reflection, electron spin resonance (ESR), nuclear magnetic resonance (NMR) and X-ray crystallography has been helpful in characterizing protein and membrane structure [7,8]. Unfortunately, the mechanism(s) and structural consequences of membrane association remain poorly understood [9,10].

In previous papers, we have used a purpose-written matrix-based computational program to predict potential lipid interfaces for several key cytoskeletal proteins (alpha-actinin, Arp2, CapZ, talin, and vinculin) [11]. Although there is no direct biochemical evidence to support the CapZ sites, the locations proposed for alpha-actinin, Arp2, talin, and vinculin are supported by *in vitro *experiments, including hydrophobic labeling, differential scanning calorimetry, film balance, T-jump, CD spectroscopy, and isothermal titration calorimetry [12-16]. In this paper we correlate the results of our predictive algorithm with the respective high-resolution three-dimensional crystal structures.

## Method

Our algorithm for predicting a protein's lipid interface identifies highly hydrophobic or amphipathic amino acid segments while discriminating between surface-seeking and transmembrane configurations [11,17-19]. An amphipathic helix, defined as an alpha- helix with opposing polar and nonpolar surfaces oriented along its long axis, is a common secondary structural motif that reversibly associates with lipids and displays detergent properties. Based on analysis of the lipid-binding properties of apolipoproteins, polypeptide hormones and lytic polypeptides, we designed our algorithm to classify amino acids into five physiochemical groups (hydrophobic, polar, positive, negative and neutral) and divide amphipathic helices spatially into three sectors (hydrophobic, interface and polar). The composition of an idealized amphipathic helix is mathematically defined by a matrix motif (M_ij_) consisting of five rows (representing the physiochemical groups) with the number of columns equal to the number of residues within the idealized helix. A comparison matrix (C_ik_) is calculated by multiplying together the matrix motif (M_ij_) and a second matrix determined for a segment of residues from the test protein (S_jk_). Summation over all components of C_ik _generates a consensus score that estimates the compatibility between a given amino acid segment and the amphipathic motif. Higher scores indicate increasing probabilities that the residues of a segment do not form an amphipathic structure by chance. The algorithm generally identifies several candidate sites per protein species.

In this study, the computationally predicted lipid-binding sites for alpha-actinin, Arp2, CapZ, talin, and vinculin are examined in the context of the respective high-resolution three-dimensional coordinates obtained from the Protein Data Bank (Tables 1, 2, 3) [20]. Qualitative graphical analysis, performed with the display programs SPDBV and PYMOL, include examination of secondary and tertiary structure, solvent accessibility and electrostatic field potentials [21,22]. The electrostatic calculations were performed by SPDBV subroutines using the Coulomb method with the dielectric constants for the solvent and protein set to 80.0 and 4.0, respectively, and incorporating only charged residues.

**Table 1:** Characteristics of the three-dimensional structures. Coordinate files were obtained from the Protein Data Bank [20]; 1HCI [28]; 1K8K [49]; 1IZN [61]; 1MIX [83]; 1MIZ [83]; 1QKR [93]; 1TR2 [92]; 1ST6 [94].

**#**	**Protein**	**Crystal**	**Organism**	**Sequence Included**	**Resolution (Å)**	**Refinement (R-value)**	**PDB ID**
1	α-actinin	Rod domain: spectrin-like repeats 1–4	Homo sapiens	274–746	2.8	0.270	1HCI
2	Arp2	Arp2/3 complex	Bos taurus	154–343^1^	2.0	0.216	1K8K
3	CapZβ-1	CapZ	Gallus gallus	2–271	2.1	0.222	1IZN
4	Talin	FERM domain (subdomains 2 and 3)	Gallus gallus	196–400	1.75	0.199	1MIX
		FERM domain/Integrin β3 tail fragment (739–743) Complex	Gallus gallus	200–400	1.9	0.204	1MIZ
5	Vinculin	Tail Domain	Gallus gallus	881–1061^2^	1.8	0.200	1QKR
		Full length (Selenium-methionine derivative)	Homo sapiens	1–1066	2.85	0.251	1TR2
		Full length	Gallus gallus	1–1065	3.1	0.316	1ST6

• ^1^Subdomains 1 and 2 are partially disordered and not included in the refined model.

• ^2^Residues 856–874 could not be adequately modeled or refined and are not included in the PDB coordinates.

**Table 2:** Computationally determined sites of probable lipid binding. A matrix algorithm [11] was used to identify probable lipid-binding sites in the following cytoskeletal proteins; α-actinin [14], Arp2 [16], CapZβ-1 (submitted, TBMM), Talin [12-13, 121] and Vinculin [14]. *In-vitro *experimental support for the computationally predicted sites for α-Actinin, Arp2, Talin, and Vinculin (site 935–978) was obtained from a variety of techniques including hydrophobic labeling, differential scanning calorimetry (DSC), Langmuir Blodgett (film balance), T-jump, CD spectroscopy, cryo-electron microscopy (EM), FTIR, and isothermal titration calorimetry.

**Protein**	**Sequence Residues**	**Species**	**Sequence**	**Experimental (*in-vitro*) Validation**
α-actinin	281–300	Gallus gallus	EKLASDLLEWIRRTIPWLEN Residues (287–306) of 1HCI	DSC, Centrifugation, SDS-PAGE [14]
	720–739	Gallus gallus	QLLTTIARTINEVENQILTR Residues (726–745) of 1HCI	DSC, Centrifugation, SDS-PAGE [14]
Arp2	185–202	A. castellanii	RDVTRYLIKLLLLRGYVF	DSC, Film Balance, Temperature Jump [16]
CapZβ-1	134–151	Homo sapiens	IKKAGDGSKKIKGCWDSI	No data
	215–232	Homo sapiens	RLVEDMENKIRSTLNEIY	No data
Talin	385–406	M. musculatus	GEQIAQLIAGYIDIILKKKKSK	Isothermal Titration Calorimetry, Monolayer Expansion, CD-spectroscopy [15]; FTIR [86] Resonance energy transfer, Cryo-EM [90]
Vinculin	935–978	Gallus gallus	RLVRGGSGNKRALIQCAKDIAKASDEVT RLAKEVAKQCTDKRIR	Co-sedimentation, Hydrophobic Photolabeling [102]
	1020–1040	Gallus gallus	TEMLVHNAQNLMQSVKETVRE	No data
	1052–1066	Homo sapiens	AGFTLRWVRKTPWYQ	No data

**Table 3:** Characteristics of sequences implicated in lipid binding. The isolelectric point for the isolated peptide was calculated and the percent alpha-helix determined from the relevant crystal structure. The symbols for electrically positive residues are underlined (); those corresponding to electrically negative residues are underlined (). The characters under the amino acid sequence refer to the secondary structure; H = helix, T = hydrogen-bonded turn, S = bend, E = extended beta-strand, and B = residue in isolated beta-bridge. Residues 401–406 (KKKKSK) are not present in talin crystal structures. Helical residues are underlined ().

**Protein**	**Residues**	**Sequence**	**Number Residues**	**Isoelectric Point**	**Helix Content**	**Sequence Site in Protein**
α-actinin	281–300		20	4.49	15/20 (75%)	Helices 1–2
	720–739		20	4.66	16/20 (80%)	Carboxyl-terminal portion of Helix 16
Arp2	185–202		18	10.0	13/18 (72%)	Helix 1 of Actin-like Subdomain 4
CapZβ-1	134–151		18	9.62	0/18 (0%)	Contains portion of β strand 6
	215–232		18	4.49	18/18 (100%)	Helix 5
Talin	385–406		22	8.61	9/22 (41%)	Helix 5 of Subdomain F3 of Talin-H
Vinculin	935–978		44	9.73	31/44 (70%)	Domain 5, Helices 2–3 + amino-terminal portion of Helix 4
						
	1020–1040		21	4.47	20/21 (95%)	Domain 5, Helix 5
	1052–1066		15			Hairpin [122]

## Results

### Alpha-actinin

Dynamic turnover of the actin network drives cell motility and muscle contraction. Alpha-actinin, one of several actin-binding proteins essential for cytoskeletal function, is a ubiquitous protein that cross-links actin filaments in muscle and non-muscle cells [23-27]. The protein is found at cell adhesion sites, focal contacts, and along actin stress-fibers in migrating cells. Alpha-actinin can localize to the plasma membrane, where it cross-links the cortical actin, aids in membrane displacement, and links transmembrane receptors with the cytoskeleton. Alpha-actinin is the major thin filament cross-linking protein in the muscle Z-discs. Mutations to the *Drosophila melanogaster *alpha-actinin gene disrupt the Z-discs and are generally lethal [26]. Translocation of alpha-actinin from the cytosol to the plasma membrane may occur indirectly by interactions with the cytoplasmic tails of transmembrane receptors. Alpha-actinin associates with several plasma membrane associated proteins including ICAM-1, ICAM-2, beta1-integrin, beta2-integrin, L-selectin, vinculin, and zyxin. The peptides that interact with alpha-actinin tend to be basic, alpha-helical, and appear to interact with the conserved acidic surface of the alpha-actinin rod [28].

Alpha-actinin may interact with phospholipid membranes directly [29]. Static light scattering experiments, employing monolayers and bilayers of varied charge composition, demonstrate that alpha-actinin reconstitutes into the hydrophobic core of lipid bilayers containing negatively charged phospholipids [30]. Phosphoinositides, such as phosphatidylinositol 3,4,5-trisphosphate (PIP_3_) and phosphatidylinositol 4,5-bisphosphate (PIP_2_), differentially regulate alpha-actinin flexibility and function [27,31-34]. Binding of phosphoinositides to alpha-actinin occurs through the calponin homology domain and has been localized to amino acids 168–184 of striated muscle species [32]. Phosphatidylinositol 3-kinase may directly bind to alpha-actinin through its p85 subunit [35]. In the presence of diacylglycerol and palmitic acid, alpha-actinin can form microfilament-like complexes with actin [36].

**Figure 1:**
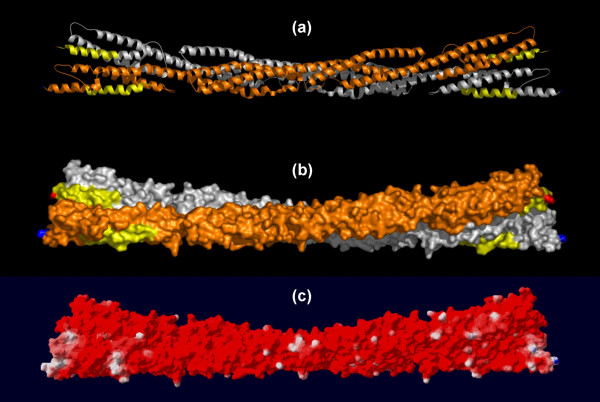
**The predicted lipid-binding site of the alpha-Actinin dimer**. The coordinates of the alpha-actinin rod domain (PDB 1HCI) are displayed with one monomer of the dimer shown in silver and the other in gold. The predicted lipid-binding sites are colored yellow. Amino and carboxyl termini are indicated in blue and red, respectively. (a) Ribbon model, (b) Space-filling representation, and (c) Electrostatic field potentials (orientation of the protein is identical to that viewed in (a) and (b)). The colors red, white and blue are used to indicate negative, neutral and positive field potentials (c), respectively.

Alpha-actinin is an anti-parallel homodimeric rod with extensive homology to spectrin and dystrophin [28,37]. The 30–40 nm long dimer consists of two identical polypeptide chains, divided into three functional domains: an actin-binding region at the amino-terminus, a central alpha-actinin segment (rod), and a carboxyl-terminus containing two EF hands (generally a 12 residue loop flanked on both sides by a 12 residue alpha helix) (Figure 1). The actin-binding region contains the amino terminal calponin-homology (CH) domain and the carboxyl-terminal calmodulin-homology (CaM) domain. The relatively rigid central rod domain (242 × 31–49 Å), derived from four spectrin repeats, defines the distance between cross-linked actin filaments and mediates interactions with receptors and signaling proteins.

Electron and cryo-electron microscopy have provided low-resolution (15 Å) images of the intact alpha-actinin molecule [38,39]. Unfortunately, only the rod domain (residues 274–746, Table 1) has been successfully crystallized for high-resolution structural studies [28]. The segments implicated in lipid-binding by our algorithm, amino acid residues 281–300 (1st spectrin repeat) and residues 720–739 (4th spectrin repeat), lie at the head/tail junctions of opposite ends of the isolated monomer in the crystallized rod domain (Figure 1; Table 2) [14]. The site experimentally implicated in phosphatidylinositide binding, amino acids 168–184, is absent from the crystallized construct [28,31]. This segment was not identified as a lipid-binding candidate by our computer algorithm, presumably because the amino acid sequence (TAPYRNVNIQNFHLSWK) forms an extended loop or coil [40].

In the dimeric rod, the predicted lipid-binding regions from constituent monomers lie close, but not confluent, to one another. The left-handed ninety-degree trans-rod twist places the dimer's two amino-terminal lipid-binding segments, residues 281–300, on a common face while separating the carboxyl-terminal segments. Amino acid residues 281–300 and 720–739 are largely alpha-helical and solvent exposed. Whether this accessibility is maintained in the intact alpha-actinin molecule is not clear from the low-resolution structural studies since the region of the protein that joins the 47 kDa head to the rod domain appears to be quite flexible [38].

Alpha-actinin is an acidic protein with a pI of 6.0. Membrane binding is not calcium-dependent but the protein may undergo conformational changes in response to salts, cations, and lipids [30,41]. The native alpha-actinin rod is globally electrostatically negative; however, the ends containing the predicted lipid-binding sites are less acidic than the middle core (Figure 1, panel c). This suggests that the dimer ends would be the most likely candidates to interact with the negatively charged phospholipids at the bilayer interface. The relatively low isoelectric points of the computationally predicted sites (Table 3) and the preponderance of surrounding negative charge in the intact rod implies a relatively weak attraction between alpha-actinin and negatively charged phospholipids in the absence of neutralizing cofactors or a significant conformational change. Surprisingly, not only do the isolated computationally identified lipid-binding fragments readily insert into lipid aggregates, but intact smooth muscle alpha-actinin preferentially binds *in-vitro *to membranes containing negatively charged phospholipids [30].

### Arp2

Arp2 (actin-related-protein), in a complex with six other proteins including Arp3, promotes branched growth of actin filaments. Immunoelectron microscopy localizes the Arp2/3 complex to the Y-branch, the point where a daughter actin filament branches off at a seventy-degree angle from the parent filament [42-44]. The Arp2/3 complex attaches to the side of the parent actin filament through the interactions between three of its five ancillary proteins (p16, p34 and p40) and actin subunits. Activation of the Arp2/3 complex requires the presence of nucleation-promoting factors and a pre-existing filament [45,46]. Nucleation factors such as WASP/Scar (Wiskott-Aldrich Syndrome), in turn, require activation through chemotactic signaling pathways that guide cellular movement. WASP promotes the binding of the Arp2/3 complex to the side of a pre-existing filament and may transfer the first actin subunit to the nascent filament's rapidly growing barbed end. Vinculin may also bind to the Arp2/3 complex, in a phosphatidylinositol-dependent manner, during membrane protrusion [47].

**Figure 2:**
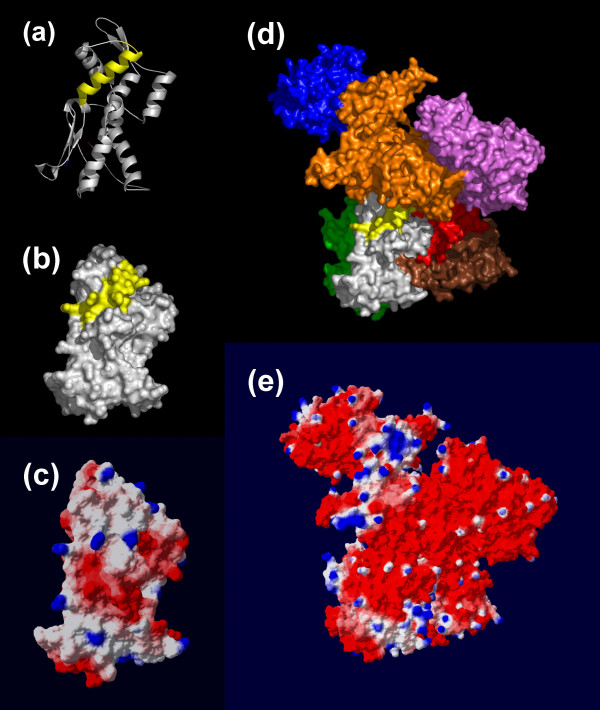
**The predicted lipid-binding site of Arp2 and the Arp2/3 complex**. The coordinates of subdomains 3 and 4 of Arp2 (PDB 1K8K) are displayed as they appear in the inactive crystallized Arp2/3 complex. The predicted lipid-binding site is colored yellow. Amino and carboxyl termini are indicated in blue and red, respectively. Arp2 subdomains 3 and 4; (a) Ribbon model, (b) Space-filling representation, and (c) Electrostatic field potentials (orientation of the protein is identical to that viewed in (a) and (b)). The crystallized Arp2/3 complex is shown as; (d) Space-filling representation (Arp2 (white), Arp3 (gold), p21 (blue), p40 (green); p34 (purple); p20 (red), p16 (brown)), and (e) Electrostatic field potentials (orientation of the protein is identical to that viewed in (d)). The colors red, white and blue are used to indicate negative, neutral and positive field potentials (e), respectively.

The Arp2/3 complex is a 220 kDa stable assembly of two actin-related proteins and five novel protein subunits [48,49]. Arp protein sequences are homologous to actin, and subunit p40 (gene name *ARPC1*) resembles a beta-propeller protein. The other 4 subunits of the complex (gene names *ARPC2 *through *ARPC5*) share little sequence homology to known proteins. The maximum dimensions of the complex are 150 × 140 × 100 Å (Figure 2) [49].

The low-resolution 'kidney bean' structure revealed for the Arp2/3 complex by electron microscopy is in general agreement with the inactive crystallographic complex [48,49]. It is thought that ATP binding induces a modest rigid body rotational conformational change, together with a more dramatic translation, that activates the Arp2/3 complex (Figure 2, panel d) [48,49]. Unfortunately, since the electron densities for subdomains 1 and 2 of Arp2 are weak, preventing accurate refinement of this region, the three-dimensional coordinates available from the Protein Data Bank are a synthesis of refined structure and molecular modeling. Subdomains 1 and 2 are modeled by the polyalanine trace of the highly homologous protein actin. Subdomains 3 and 4 of Arp2, which are adequately visualized and refined, also resemble actin.

Our algorithm predicts that amino acid residues 185–202 of Arp2 are involved in mediating lipid interactions. The isolated segment partially inserts into lipid aggregates with an apparent K_d _of 1.1 μM [16]. In the crystal structure, this segment is primarily alpha-helical (72 %) and lies near the center of the Arp2/3 complex (Figure 2, panel d) [49]. The helix is relatively recessed within Arp2 and solvent access is further limited by the presence of adjacent proteins in the complex. It is likely that subdomains 1 and 2 of Arp2, which are missing from the refined structure, would further limit the ability of residues 185–202 to interact directly with lipids in the absence of a substantial rearrangement of the ternary complex.

Both p21 and p40 have substantial areas of positive surface charge. These regions are relatively remote from the Arp2's predicted lipid interface in the inactive complex. The computationally predicted lipid interaction site is itself electrostatically neutral but surrounded by strong negative potentials in the assembled complex (Figure 2, panel e). Thus, the interaction of Arp2 with lipids is likely to occur either prior to assembly of the complex or after a significant conformational change (as postulated for activation) that reduces local charge barriers and improves solvent access.

### CapZ β1

Capping protein is crucial for actin filament assembly. Activated Cap binds to the barbed end of actin with high affinity (K_d _= 1nM) and at a 1:1 stoichiometry forming a mechanical 'cap' that prevents the addition or loss of actin monomers [50,51]. The sarcomeric isoform of capping protein, which is composed of two polypeptide chains (CapZ α1-β1), localizes to the Z-line of muscle through an interaction with alpha-actinin [52]. The non-sarcomeric isoforms are localized at the sites of membrane-actin contact [53-56]. Capping protein 'caps' the Arp1 mini-filament in the dynactin complex, directly interacts with twinfillin, and indirectly affects the Arp2/3 complex via the CARMIL protein [57-60]. Residues at the carboxyl-termini of each CapZ chain (α 259–286 and β 266–277) are essential for actin binding.

**Figure 3:**
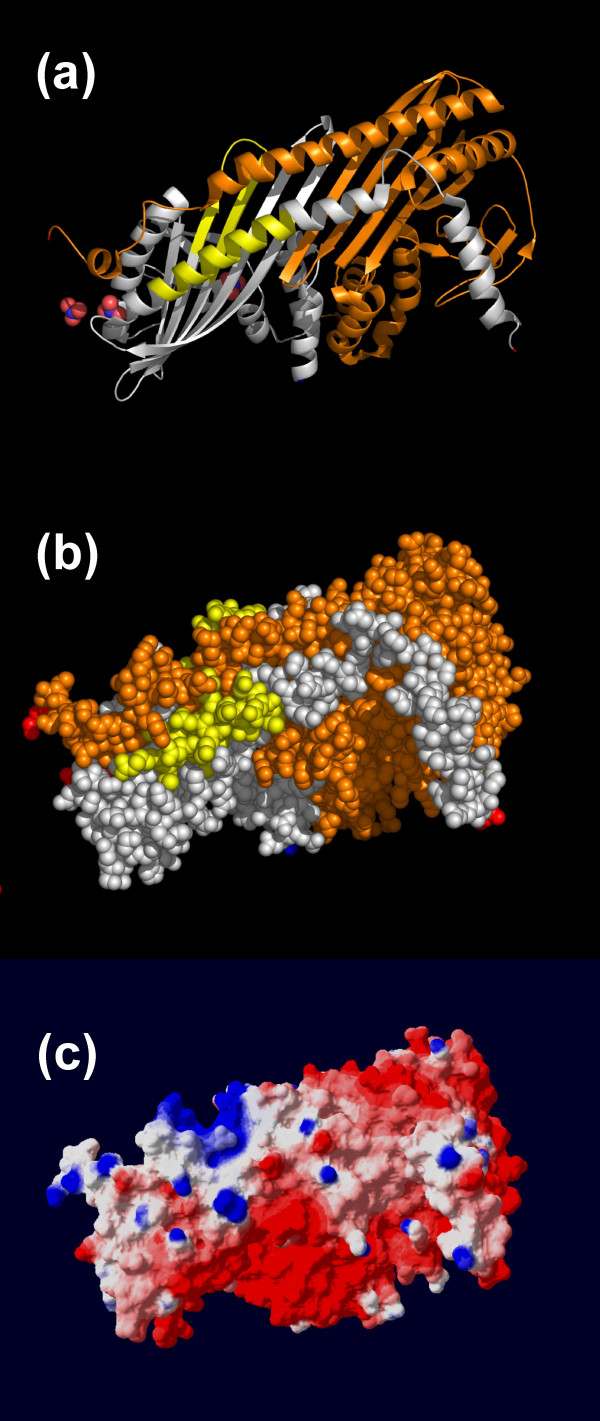
**The predicted lipid-binding site of CapZbeta-1**. The coordinates of CapZ (PDB 1IZN) are displayed with the alpha subunit shown in gold and the beta subunit in silver. The predicted lipid-binding sites are colored yellow. Amino and carboxyl termini are indicated in blue and red, respectively. (a) Ribbon model, (b) Space-filling representation, and (c) Electrostatic field potentials (orientation of the protein is identical to that viewed in (a) and (b)). The colors red, white and blue are used to indicate negative, neutral and positive field potentials, respectively.

CapZ is an elongated, tightly assembled, heterodimeric alpha/beta protein with overall dimensions of 90 × 50 × 55 Å [61]. The two subunits, which may have arisen from gene duplication, are structurally homologous creating a pseudo two-fold symmetry perpendicular to the long axis of the molecule (Figure 3). Each subunit contains three domains and an additional carboxyl-terminal extension. Three anti-parallel helices in an up-down-up arrangement (helices 1–3) form the amino-terminal domain. The middle domain is composed of four beta strands (strands 1–4 for the alpha subunit; three beta strands 1–3 for the beta subunit), containing two reverse turns. The carboxyl-terminal domain comprises an anti-parallel beta sheet formed by five consecutive beta strands (strands 5–9), flanked on one side by a short amino-terminal helix (helix 4) and a long carboxyl-terminal helix (helix 5). The beta strands of each subunit form a single 10-stranded anti-parallel beta-sheet in the center of the molecule. A 'jellyfish' model has been proposed for Cap function in which the carboxyl-terminal helical regions of the protein are mobile and extend outward to engage the barbed end of actin [61].

Phosphatidylinositol 4,5-bisphosphate (PIP_2_) regulates CapZ function by dissociating the protein from the barbed ends of actin filaments [59,62]. This effect appears to be due to the direct binding of dispersed PIP_2 _to CapZ. High concentrations of other anionic phospholipids also inhibit the ability of CapZ to effect actin polymerization [63]. In some phosphatase and kinase structures, nitrate ions have been found near the phosphate binding sites mimicking the transition state [64-68]. Sulfate ion also may serve as a marker for phospholipid binding sites. The crystal structure of CapZ beta-1 contains four nitrate ions [61]. Only two nitrate ions appear to bind to the protein with high specificity; one nitrate is associated with Lys95 while the other interacts with the dipole of helix 5 (Figure 3, panel a). These nitrate-binding sites, located near the actin-binding carboxyl-terminal extension of the Z subunit, suggest a potential mechanism for PIP_2 _regulation of CapZ – actin association.

The sequences predicted to mediate lipid binding by our algorithm, amino acid residues 134–151 and 215–232 of the CapZ-β1 subunit, lie adjacent to one another in the crystal structure [61]. Residues 134–151 primarily form beta-sheet whereas residues 215–232 are part of Helix 5. Both segments are solvent-accessible despite contributing residues to the strong dimer interface (e.g., Lys136, Glu221 and Asn222). Although CapZ is predominantly electrostatically negative, the proposed lipid-binding interface varies from neutral to positive (Figure 3, panel c).

### Talin

Talin is an abundant cytoskeletal protein that binds to the cytoplasmic tails of integrin beta subunits, to actin filaments, to other actin-binding proteins, and to phospholipids [12,69-76]. In fibroblasts, the binding of talin to membranes may induce the formation of focal adhesions or trigger actin assembly by activating integrins or layilin, respectively. In platelets, activated talin translocates from the cytoplasm to the membrane where it co-localizes with the GPIIb/IIIa complex [76].

Talin is a member of the 4.1 superfamily of FERM proteins, a group of membrane-associated proteins that includes the erythrocyte membrane protein 4.1, the ezrin, radixin, moesin, and merlin proteins, and some tyrosine phosphatases [77]. A common feature of FERM domain proteins is extensive intramolecular head-tail interactions that mask binding sites on the head [78,79]. Association of extracellular matrix ligands with integrins triggers the binding of the second messenger phosphatidylinositol 4,5-bisphosphate (PIP_2_) to the head domain, altering its conformation to allow talin to bind to the cytoplasmic tails of integrin receptors [78]. Binding occurs through a largely hydrophobic area centered on the b5 strand and also involves residues of the b6 strand, the carboxyl-terminal half of helix H5 and the b4-b5 loop. During outside-in integrin signaling, talin binds to other partners on the cytoplasmic face of adhesion complexes, and in particular vinculin, which then binds directly to actin and induces actin bundling [80,81]. The incorporation of talin into zwitterionic phospholipid bilayers is low but improved in the presence of negatively charged phospholipids (K = 2.9 × 10^6 ^M^-1^) [13]. Talin is able to bind *in vitro *to phosphatidylinositol, phosphatidylinositol 4-monophosphate, and PIP_2_. However, within a phospholipid bilayer, binding is restricted to PIP_2_.

**Figure 4:**
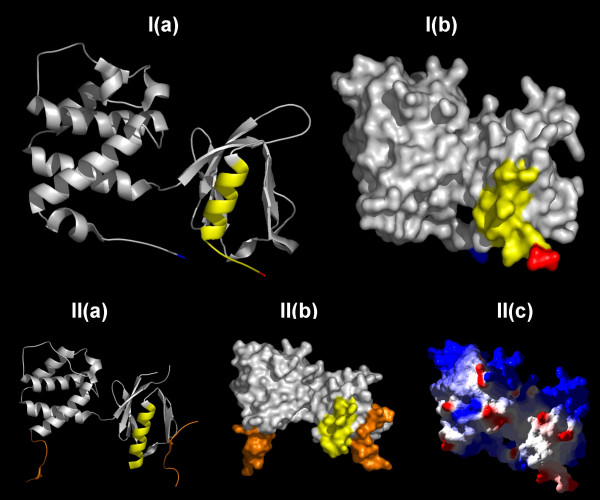
**The predicted lipid-binding site of Talin**. The coordinates of talin are displayed either in (I) isolation (1MIX); or (II), in a complex with an integrin beta3 tail fragment (residues 739–743) (1MIZ). The predicted lipid-binding sites are colored yellow and the integrin beta3 tail fragment gold. Amino and carboxyl termini are indicated in blue and red, respectively. (a) Ribbon model, (b) Space-filling representation, and (c) Electrostatic field potentials (orientation of the protein is identical to that viewed in (a) and (b)). The colors red, white and blue are used to indicate negative, neutral and positive field potentials (c), respectively.

Talin is a flexible 235 kDa 51 nm dumbbell-shaped homodimer (Figure 4) [82,83]. Calpain cleavage before amino acid residue 434 yields 2 major domains, an N-terminal 47 kDa FERM head and a carboxyl 190-kDa rod domain. The rod domain, which is responsible for actin interaction and nucleation, contains low-affinity integrin binding sites as well as actin and vinculin binding sites [84,85]. The isolated 47 kDa FERM-containing domain retains the lipid-binding capacity of intact talin and includes a primary integrin-binding site [71]. Talin binds to phospholipids using both hydrophobic and electrostatic forces with a strong preference for negatively charged aggregates [86].

FERM domains are cysteine-rich modules that bind phosphoinositides via amino acid sequences with a high percentage of basic and polar amino acids. FERM domains contain three modules arranged in a clover shape: F1, F2 and F3 [87]. The F3 module of talin, which structurally resembles a phosphotyrosine-binding domain, is formed by a single carboxyl-terminal helix that partly encloses one edge of an internally hydrophobic beta sandwich [88]. A consensus sequence for PIP_2 _binding has been described (K/R)XXXKX(K/R)(K/R) but exceptions are frequent [89].

The computationally predicted lipid-binding site, amino acids 385–406, has a calculated hydrophobicity of 0.029, high amphipathicity, and a hydrophobic moment of 0.3 [13,15,90]. At pH 7.4 the total free energy of binding (ΔG_0_) is approximately -9.4 kcal/mol, a value that compares favorably with that determined for myristylolated membrane-anchoring peptides. Residues 385–406 lie within helix 5 and thus contribute substantially to the binding site for the integrin beta3 tail. This proximity suggests a mechanism for the PIP2 induced conformational change that permits tail binding [78].

### Vinculin

Vinculin is a conserved regulator of cell-cell adhesion (cadherin-mediated) and cell-matrix focal adhesions (integrin/talin-mediated). In its resting state, vinculin is held in a closed conformation through interactions between its head (Vh) and tail (Vt) domains. Vinculin activation, associated with junctional signaling, generates an open conformation that binds *in vitro *to talin, alpha-actinin, paxillin, actin, the Arp2/3 complex, and to itself [47,91-95].

Talin and phospholipids activate vinculin. Talin binds to Vh through high-affinity vinculin-binding sites present in its central rod domain. Talin binding stimulates conformational changes in the amino-terminal helical bundle of Vh, displacing the tightly bound Vt [95]. Talin also increases the activity of phosphatidylinositol phosphate kinase-1 γ, generating PIP_2 _[96-99]. The binding of phosphatidylinositol 4,5-bisphosphate to Vt, in turn, disrupts the Vh-Vt interaction freeing vinculin to bind talin, actin, VASP or the Arp2/3 complex [100]. Vinculin can readily insert into the hydrophobic core of mono/bilayers containing acidic (phosphatidic acid, phosphatidylinositol and phosphati-dylglycerol), but not neutral (phosphatidylcholine and phosphatidylethanolamine), lipids [101,102]. Vinculin can also undergo covalent modification by lipids *in vivo *or bind acidic phospholipids through its carboxyl-terminal domain (amino acids 916–970) [103-106]. The latter process may inhibit the intramolecular association between the amino and carboxyl terminal regions of vinculin and/or expose a binding site for protein kinase C [107,108].

**Figure 5:**
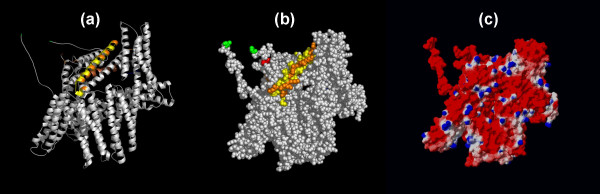
**The predicted lipid-binding site of Vinculin**. The coordinates of vinculin (PDB 1ST6) are displayed with the predicted lipid-binding sites colored yellow (residues 935–978) and brown (residues 1020–1040). Phosphatidylinositol 4,5-bisphosphate appears to bind to a basic "collar" surrounding the carboxyl-terminal arm (residues 910, 911, 1039, 1049, 1060, 1061), and a basic 'ladder' along the edge of helix 3 (residues 944, 945, 952, 956, 963, 966, 970, 978, 1008, and 1049). These residues are shown in gold. *Note: *the overlap of the computationally derived site and the experimentally discovered phosphatidylinositol site. Amino and carboxyl termini are indicated in blue and red, respectively. Residues 856 through 874 are disordered in the vinculin electron-density map and are not shown, the start (residue 855) and stop site (residue 874) for this region are shown in green. (a) Ribbon model, (b) Space-filling representation, and (c) Electrostatic field potentials (orientation of the protein is identical to that viewed in (a) and (b)). The colors red, white and blue are used to indicate negative, neutral and positive sfield potentials (c), respectively.

Vinculin is a large (1,066 amino acid), structurally dynamic protein with overall dimensions of 100 × 100 × 50 Å in its autoinhibited conformation (Figure 5) [92-95]. The protein is composed of eight four-helix bundles that divide the protein into five distinct domains; an 850 amino acid head (Vh), a 200 amino acid tail (Vt) and 3 intervening linkers (Vh2, Vh3, Vt2). The sequences implicated in lipid binding by our algorithm, amino acid residues 935–978 and 1020–1040, contribute to helices 2 through 5 of Vt. Segment 935–978 includes residues involved in Vt-Vh interactions (Arg 945, Arg 978) as well as those mediating phosphatidylinositol binding. Phosphatidylinositol 4,5-bisphosphate appears to bind to a basic "collar" surrounding the carboxyl-terminal arm (residues 910, 911, 1039, 1049, 1060 and 1061), and a basic 'ladder' along the edge of helix 3 (residues 944, 945, 952, 956, 963, 966, 970, 978, 1008 and 1049) (Figure 5, panel a). Point mutations in the collar (Lys911Ala and Lys924Ala) or ladder (Lys952Ala) reduce PIP_2 _binding by 50%. The ladder is largely solvent exposed, although at its amino-terminal end Lys944 and Arg945 make salt bridges to acidic residues on the head. His906, which lies adjacent to the computationally predicted lipid-binding site, is essential for PIP_2 _induced conformational changes [110]. Binding of 10% PIP_2 _in phosphatidylcholine vesicles to Vt occurs in the micromolar range, but in combination with PIP_2 _miscelles and talin, vinculin appears to form a ternary activation complex.

## Discussion

Intracellular signaling and trafficking are regulated by selective protein-membrane interactions. Transfer of cytosolic proteins to the membrane presumably occurs in two steps: an initial approach based on electrostatic attraction followed by lipid-induced protein refolding and/or insertion [110]. Potential control mechanisms include: (1) modulating the protein's affinity for lipid (e.g., calcium-binding promotes the membrane association of C2 domains by enhancing electrostatic forces), (2) sequestering the lipid at specific locations, and/or (3) restricting access to the lipid in the absence of specific stimuli [10,111-113].

*In-vitro *experimental support for the computationally predicted lipid-binding sites of α-Actinin, Arp2, Talin, and Vinculin (site 935–978) was obtained using standard techniques such as hydrophobic labeling, differential scanning calorimetry (DSC), Langmuir Blodgett (film balance), FTIR, T-jump, CD spectroscopy, cryo-electron microscopy (EM), and isothermal titration calorimetry. Similar data are not yet available to gauge the *in-vitro *binding characteristics of the sites predicted by our algorithm for CapZbeta-1 or the vinculin sites (residues 1020–1040 and 1052–1066).

The three-dimensional structures of the computationally predicted lipid-binding sites described here are, with the exception of Site 1 of CapZbeta-1, predominantly or exclusively alpha-helical. The energy required to insert a polypeptide into a membrane is minimized by the presence of favorable secondary structure [114]. Membrane-spanning or surface associated amphipathic alpha-helices and beta-strands/sheets are common in biologically active peptides and proteins. Amphipathic alpha-helices may reversibly associate with lipids and function as peptide detergents [115-117]. Amphipathic beta-sheets, in contrast, interact with lipids in an essentially irreversible manner, and lack detergent properties. Unfavorable energy costs associated with individual amphipathic beta-strands are likely to drive coalesence into beta-sheets on lipid surfaces. When the axis of an amphipathic helix lies parallel to the membrane surface and partially inserted into the membrane, the polar and non-polar protein surfaces may interact simultaneously with the charged head groups and hydrophobic side chains, respectively.

Four of the five cytoskeletal proteins studied here show a strong preference for acidic phospholipids *in vitro *(alpha-actinin, Arp2, talin, and vinculin). The mechanism by which these soluble cytoplasmic proteins become membrane associated is unclear. Only one of the five proteins is known to undergo covalent lipid modification (i.e., vinculin). Although myristoylation and palmitoylation increase hydrophobicity, myristate alone may be insufficient to anchor proteins to the plasma membrane [1,118,119]. The clustering of basic residues adjacent to lipid modification sites found among proteins such as K-ras4B and HIV-1 Gag enhances favorable electrostatic interactions with acidic lipids [19,66]. Other peripheral proteins (e.g., type II beta-phosphatidyinositol-3-kinase, AKAP79, myelin basic protein, and a number of proteins containing C2 domains), in the absence of lipophilic modifications, depend solely upon basic groups to bind to membrane surfaces [112]. The three-dimensional structures of pleckstrin homology domains reveal large positively charged electrostatic patches surrounding the ligand-binding sites, suggesting that the excess charge is useful in improving initial attraction and orientation to the predominantly negatively charged plasma membrane [113]. Most of the predicted lipid interface sites in this study are either intrinsically electrostatically positive (Table 3) or are located in regions that are relatively basic.

Many critical biological pathways are regulated by protein-lipid interactions. Understanding this biology is difficult given the complexity and heterogeneity of the interface. Computational methods, such as our matrix algorithm, provide a potentially powerful means for predicting the region, and orientation, of a protein as it associates with lipid aggregates. Further experimental work will be required to validate and refine this algorithm. However, based on experience with the protein huntingtin, it appears that the methods may be applicable to multiple protein classes [120].
